# Co-Expression of SERCA Isoforms, Phospholamban and Sarcolipin in Human Skeletal Muscle Fibers

**DOI:** 10.1371/journal.pone.0084304

**Published:** 2013-12-16

**Authors:** Val A. Fajardo, Eric Bombardier, Chris Vigna, Tahira Devji, Darin Bloemberg, Daniel Gamu, Anthony O. Gramolini, Joe Quadrilatero, A. Russell Tupling

**Affiliations:** 1 Department of Kinesiology, University of Waterloo, Waterloo, Ontario, Canada; 2 Department of Physiology, University of Toronto, Toronto, Ontario, Canada; University of Minnesota, United States of America

## Abstract

Sarcolipin (SLN) and phospholamban (PLN) inhibit the activity of sarco(endo)plasmic reticulum Ca^2+^-ATPases (SERCAs) by reducing their apparent affinity for Ca^2+^. A ternary complex between SLN, PLN, and SERCAs results in super-inhibition of SERCA activity. Analysis of skeletal muscle homogenate has limited our current understanding of whether SLN and PLN regulate SERCA1a, SERCA2a, or both in skeletal muscle and whether SLN and PLN are co-expressed in skeletal muscle fibers. Biopsies from human vastus lateralis were analyzed through single fiber Western blotting and immunohisto/fluorescence staining to circumvent this limitation. With a newly generated SLN antibody, we report for the first time that SLN protein is present in human skeletal muscle. Addition of the SLN antibody (50 µg) to vastus lateralis homogenates increased the apparent Ca^2+^ affinity of SERCA (*K*_Ca_, pCa units) (-Ab, 5.85 ± 0.02 vs. +Ab, 5.95 ± 0.02) and maximal SERCA activity (μmol/g protein/min) (-Ab, 122 ± 6.4 vs. +Ab, 159 ± 11) demonstrating a functional interaction between SLN and SERCAs in human vastus lateralis. Specifically, our results suggest that although SLN and PLN may preferentially regulate SERCA1a, and SERCA2a, respectively, physiologically they both may regulate either SERCA isoform. Furthermore, we show that SLN and PLN co-immunoprecipitate in human vastus lateralis homogenate and are simultaneously expressed in 81% of the fibers analyzed with Western blotting which implies that super-inhibition of SERCA may exist in human skeletal muscle. Finally, we demonstrate unequivocally that mouse soleus contains PLN protein suggesting that super-inhibition of SERCA may also be important physiologically in rodent skeletal muscle.

## Introduction

 Sarco(endo)plasmic reticulum Ca^2+^-ATPases (SERCAs) are major regulators of intracellular free Ca^2+^ in skeletal muscle, which use free energy released from the hydrolysis of ATP to transport Ca^2+^ ions from the cytosol into the lumen of the sarcoplasmic reticulum (SR) [1]. SERCAs are 110 kDa integral membrane proteins belonging to the P-type family of cation transporters, as they are phosphorylated at a critical aspartate residue during their catalytic cycle [2]. In vertebrates, there are three distinct genes (*ATP2a1*/serca1, *ATP2a2*/serca2, *ATP2a3*/serca3) that produce more than 10 isoforms through alternative splicing [3]. In adult skeletal muscle, two SERCA isoforms predominate, namely SERCA1a and SERCA2a, which are differentially expressed in specific muscle fiber types [3–6]. Specifically, the SERCA1a isoform with faster kinetics [3], is co-expressed with fast myosin heavy chain (MHC) isoforms MHCIIb, MHCIIx, and MHCIIa, while the slower SERCA2a isoform [3], is co-expressed with slow MHC isoforms MHCIβ and MHCIα [5,7]. The differences in the functional capacity of individual SERCA isoforms can be further amplified by the presence of the SERCA regulatory proteins, phospholamban (PLN) and sarcolipin (SLN) [8]. 

PLN (52 amino acid residues) and SLN (31 amino acid residues) are two functionally homologous proteins that can each regulate either SERCA1a or SERCA2a through physical interactions by lowering their apparent Ca^2+^ affinity, at least when studied in a model expression system [9,10]. Results from previous studies in skeletal muscle homogenates suggest that PLN regulates SERCA2a *in vivo* [11,12], but the results are not as clear with respect to SLN. Based on co-purification of SLN with SERCA1a from rabbit fast-twitch skeletal muscle [13], and its significantly higher mRNA expression in rabbit fast-twitch muscles compared with slow-twitch muscles [10,14], it was originally hypothesized that SLN would act as a counterpart of PLN by regulating SERCA1a in fast-twitch muscle [10]. However, more recently we found that SLN protein expression in skeletal muscles coincides with SERCA2a whereby SLN and SERCA2a levels were highest in soleus and red gastrocnemius (RG), very low in extensor digitorum longus (EDL), and undetectable in white gastrocnemius (WG) [15]. The possibility that both PLN and SLN can regulate SERCA2a *in vivo* is further justified given that both regulatory proteins are found within the atria of a variety of species [12,16] where SERCA2a is the only isoform. Therefore, we proposed that SLN is a homologue of PLN that regulates both SERCA2a and SERCA1a in a variety of muscle tissues. A major limitation with studies published to date on the expression patterns of PLN and SLN in skeletal muscle is that analyses have only been carried out on whole muscle preparations. Since both SERCA isoforms are expressed in muscles that express SLN [15] and PLN [11,17], it is not possible to say whether PLN and SLN regulate SERCA1a, SERCA2a, or both in skeletal muscle.

Another unresolved issue is whether PLN and SLN are normally co-expressed with SERCA in the same muscle fibers *in vivo*. Co-expression of PLN and SLN in HEK-293 cells results in super-inhibition of SERCA due to the formation of a ternary complex between SLN, PLN, and SERCA1a or SERCA2a that reduces affinity for Ca^2+^ as well as *V*_*max*_ [9]. This does not appear to be a problem in mouse skeletal muscle because we were not able to detect any PLN protein in mouse muscles [15]. In contrast, PLN protein is highly expressed in human skeletal muscle [11,17], but our understanding of SLN expression in humans has been limited to mRNA [14]. If SLN protein is found to be expressed in human skeletal muscle then super-inhibition may, in fact, occur physiologically. Alternatively, if expression of SLN and PLN protein is confined to different muscle fibers then super-inhibition of SERCAs could be avoided *in vivo*. 

The purpose of this study was to determine if PLN and SLN regulate SERCA1a, SERCA2a or both in human skeletal muscle. To this end, we used a newly generated antibody directed against SLN to show unequivocally the presence of functional SLN protein in human vastus lateralis and then utilized single fiber Western blotting [5], immunohisto/fluorescence staining [15,18], and co-immunoprecipitation to determine the expression pattern of PLN and SLN with respect to MHC- and SERCA- isoforms. We hypothesized that SLN would be expressed only in fast-twitch fibers with SERCA1a, whereas PLN would only be expressed in slow-twitch fibers with SERCA2a in human vastus lateralis.

## Materials and Methods

### Participants

 Five healthy untrained male university students participated in this study. The mean age, height and mass of the participants were 18.8 ± 1.0 yr, 182.4 ± 6.8 cm, and 72.5 ± 4.7 kg, respectively. As a condition of entry into the study, participants were asked to refrain from vigorous exercise, caffeine and alcohol one week prior to the collection of tissue. As required, all participants were fully informed of all experimental procedures and all associated risks before written consent was obtained. Written approval for the research was granted by the Human Research Ethics Committee at the University of Waterloo.

Muscle tissue samples (~50 -100 mg) were obtained from the vastus lateralis using the needle biopsy technique under suction [19]. One portion of the muscle biopsy sample (~30 mg) was oriented under a dissecting microscope, mounted with OCT medium, rapidly frozen in isopentane that was pre-cooled with liquid nitrogen and stored at -80°C until immunohisto/fluorescence analysis. These samples were used for the determination of fiber type specific SERCA1a, SERCA2a, and PLN expression. The remaining portion of the biopsy was cleaned of visible connective tissue, blood, and fat and then halved to be homogenized [20] for co-immunoprecipitation and SERCA activity assays or freeze-dried for single fiber Western blot analysis. 

### Antibodies

Primary antibodies against MHCI (BA-F8), MHCIIa (SC-71) and MHCIIx (6H1) [21,22] were obtained from Developmental Studies Hybridoma Bank. The primary SERCA1a antibody (A52) was a kind gift from Dr. David MacLennan (University of Toronto) [23], while SERCA2a (2A7-A1), and PLN (2D12) antibodies were obtained from Pierce Antibodies, Thermo Fisher Scientific Inc.. The primary antibody directed against SLN was generated by Lampire Biological Laboratories (PA, USA) using the C-terminus sequence LVRSYQY [24]. Secondary antibodies for Western blotting, goat anti-mouse IgG (peroxidase conjugated) and goat anti-rabbit IgG (peroxidase conjugated), were obtained from Santa Cruz Biotechnology. Secondary antibodies for immunofluorescence staining, Alexa Fluor 350 anti-mouse IgG_2b_, Alexa Fluor 488 anti-mouse IgG_1_, and Alexa Fluor 555 anti-mouse IgM, were obtained from Molecular Probes.

### Collection and preparation of single muscle fibers

A total of 509 segments of individual muscle fibers (83 - 117 per subject) were dissected from freeze-dried portions of muscle biopsies as previously described [5,25] using a dissecting microscope (Nikon SMZ1000) and fine jeweler’s forceps. A reticle (Crossline, 25mm scale, 10mm/100 div, Nikon) was used to estimate the average length (1.5 ± 0.1 mm) and diameter (55.1 ± 0.5 μm) of the fibers. With the use of forceps and small pieces of black silk sutures (4.0 USP), individual fiber segments were placed into 4 μl of 3x solubilizing buffer (1 M Tris base [pH 6.8], 6% [w/v] SDS, 30% glycerol [v/v], 15% [v/v] 2-mercaptoethanol, and 0.06% [w/v] bromophenol blue), diluted 3:1 (vol/vol) with dH_2_O and stored at -20°C until Western blotting. 

### Western blot analysis

 The validity of the newly generated SLN antibody was tested using Western blot analysis. HEK-293 cells were transfected with one of the following cDNAs: 1) NF-SLN (a fusion protein of SLN with the FLAG epitope, MDYKDDDDK, at its N terminus); 2) SERCA1a; or 3) PLN. The transfected HEK-293 cells, homogenate from mouse atria, and homogenate from human vastus lateralis were solubilized into 1x buffer and proteins were electrophoretically separated on 14% glycine gels. Following electrophoresis, proteins were transferred onto polyvinylidene difluoride (PVDF) membranes (Immobilon, Millipore, MA, USA) with transfer buffer (25 mM glycine, 192 mM Tris base, 20% methanol, 0.1% [w/v] SDS). Membranes were incubated in blocking solution (TBST buffer: 20mM Tris base, 137 mM NaCl, and 0.1% (v/v) Tween 20, pH 7.5, with 5% (w/v) non-fat dry milk) for 1 hour to block all non-specific binding sites. The membranes were then incubated for 1 hour in either 5% milk-TBST containing the newly generated SLN antibody (1:100) or containing the newly generated SLN antibody (1:100) combined with SLN blocking peptide (Lampire Biological Laboratories) in a 5:1 peptide to antibody ratio. The membranes were then washed and incubated for 1 hour in 5% milk-TBST containing goat anti-rabbit IgG (peroxidase conjugated) with a 1:2000 dilution. Membranes were washed again and antibody-antigen complexes were visualized with a Chemi Genius^2^ Bio Imaging system (Syngene, MD, USA) after addition of sensitive chemiluminescent substrate, Luminata^TM^ Forte Western HRP Substrate (Millipore, MA, USA).

Skeletal muscle fiber type co-distribution of SLN, PLN, SERCA1a and SERCA2a was also determined by Western blotting. Fiber type distribution of these proteins was determined with the co-expression of MHC isoforms, with MHCI indicating Type I fibers and MHCIIa indicating Type IIA fibers. Due to the small volume used to store single fibers (each in 12µl total volume), protein concentration prior to Western blot analysis was not determined, and no analysis of other MHCII isoforms was performed. Tricine SDS-PAGE electrophoresis was performed on gradient gels for efficient separation of all proteins on one gel [26]. Specifically, a 13% separating gel was overlaid with a layer of 6.6% separating gel and topped with 4% stacking gel. To allow for complete assessment of SERCA isoforms, MHC isoforms, PLN, and SLN, samples were halved and loaded (6 μl each) onto two separate gels (Gel A, Gel B), along with BioRad Precision Plus Western C^TM^ Molecular Weight Marker. Electrophoretically separated proteins were transferred onto two separate PVDF membranes and then blocked for 1 hour. To assess fiber type co-distribution of SERCAs, PLN, and SLN from each freeze-dried fiber segment, PVDF membranes were cut into three strips and were then incubated for 1 hour in 5% milk-TBST containing the appropriate antibodies. The first strip consisted of proteins between 150 - 250 kDa and was probed for MHCI (~200 kDa, 1:100, Membrane A) and MHCIIa (~200 kDa, 1:250, Membrane B). The second strip with proteins between 37 - 150 kDa was probed for SERCA1a (~110 kDa, 1:10 000, Membrane A) and SERCA2a (110 kDa, 1:2000, Membrane B). Finally, the third strip had proteins between 4 - 37 kDa, which was probed for PLN (5 kDa in monomeric form, 1:2000, Membrane A), and SLN (4-6 kDa, 1:100, Membrane B). The membranes were then washed and incubated for 1 hour in 5% milk-TBST-containing either goat anti-mouse IgG (peroxidase conjugated) with a 1:20000 dilution for SERCA1a, and a 1:2000 dilution for SERCA2a, PLN, MHCI, MHCII, or goat anti-rabbit IgG (peroxidase conjugated) in a 1:2000 dilution for SLN. Antibody-antigen complexes were visualized after addition of sensitive chemiluminescent substrate, Luminata^TM^ Forte Western HRP Substrate (Millipore, MA, USA) for SERCA2a, PLN, SLN, MHCI, and MHCIIa, and ECL Western Blot Substrate (BioVision, CA, USA) for SERCA1a.

### Semi-quantitative Western blot analysis

We performed semi-quantitative Western blotting on 48 single fibers (12 from 4 different subjects). To control for inter-gel variability and allow for proper analysis, 5 μg of homogenate standard from human vastus lateralis was loaded alongside the single fibers. Western blotting was carried out as described above. Optical densities for SLN, PLN, SERCA1a, and SERCA2a were obtained using GeneTools (Syngene, MD, USA) and were corrected for inter-gel variability and normalized to fiber volume that was calculated using fiber length and diameter measurements to control for the variation in protein loading.

### Co-immunoprecipitation

 Co-immunoprecipitation was used to assess the physical interactions between: 1) SERCAs-SLN, 2) SERCAs-PLN, and 3) SLN-PLN. Protein G bound to Agarose beads (Santa Cruz Biotechnology, sc-2002) were coupled with 5μg of antibodies directed against SERCA1a, SERCA2a, PLN or SLN. The Protein G agarose bead-antibody complexes were incubated overnight at 4°C with 400 μg of protein from human vastus lateralis homogenate. After low-pH elution, proteins were solubilized in 5x solubilizing buffer and then stored at -80°C until Western blot analysis, where membranes were probed for SLN, PLN, SERCA1a or SERCA2a. 

### Immunofluorescence and immunohistochemistry

 Serial cross sections of tissue (10 μm) were cut in a cryostat maintained at -20°C. Immuno-fluorescence analysis of MHC expression was previously described [18,27] and performed with primary antibodies against MHCI, MHCIIa, and MHCIIx. Briefly, cross-sections were blocked for 1 hr at room temperature (RT) using 10% goat serum followed by incubations with a mixture of primary antibodies against MHCI (1:50), MHCIIa (1:600), and MHCIIx (1:100) for 1 hr at RT. Sections were then washed (3 x 5 min) in phosphate buffered saline (PBS; 10mM, pH 7.2) and incubated with a mixture of Alexa Fluor 350 anti-mouse IgG_2b_, Alexa Fluor 488 anti-mouse IgG_1_, and Alexa Fluor 555 anti-mouse IgM secondary antibodies for 1 hr at RT. Sections were washed (3 x 5 min) in PBS and mounted with Prolong Gold antifade reagent (Molecular Probes). Slides were then visualized with an Axio Observer Z1 fluorescent microscope equipped with standard Red/Green/Blue filters, an AxioCam HRm camera, and AxioVision software (Carl Zeiss). 

 Immunohistochemistry was carried out according to the procedures previously described by Tupling et al. [28] with minor modifications. In brief, frozen muscle sections were fixed to microscope slides in a 100% acetone solution for 10 minutes at 4°C, washed (5 min) in PBS and permeabilized in 0.5% Triton X-100 in PBS for 5 min. After another wash (3 x 5 min) in PBS, all sections were blocked for 30 min at RT in a humidified chamber with a 5% horse serum solution, after which antibodies for SERCA1a (1:2500), SERCA2a (1:1000) and PLN (1:250) were applied to the individual sections for 1 hr at RT. After the sections were washed (3 x 5min) in PBS, biotinylated horse anti-mouse IgG (Vector Laboratories) was applied for 30 min at RT. Following another rinse in PBS, the sections were incubated for 30 min with a 1:500 dilution of a horseradish peroxidase-streptavidin conjugate (Vector Laboratories). SERCA1a, SERCA2a and PLN antibody binding was visualized using a horseradish peroxidase secondary detection system (NovaRED substrate kit, Vector Laboratories), which produces a brown-red precipitate. 

To determine fiber type specific protein (SERCA1a, SERCA2a, PLN) expression, a total of 1035 individual muscle fibers (124 - 272 per subject) were matched with corresponding serial sections stained for SERCA1a, SERCA2a, and PLN via a microscope (Nikon) linked to computer-based imaging analysis software (Image-Pro PLUS). The SERCA1a and SERCA2a stains were categorized into 4 intensities (dark, medium, light and blank), while PLN was only categorized into dark and light. 

### SERCA activity

 Ca^2+^-dependent Ca^2+^-ATPase activity in homogenates (n=4) was measured at 37°C as described previously [29,30]. The data were analyzed by nonlinear regression with computer software (GraphPad Software), and the *K*_Ca_ values were calculated using an equation for a general cooperative model for substrate activation. The values for maximal SERCA activity were taken directly from the experimental data and normalized for total protein concentration (μmol/g protein/min). To determine the effects of the SLN antibody on SERCA activity, 25 µL of vastus lateralis homogenate were preincubated for 30 min on ice with or without 50 µg of SLN antibody prior to initiating the SERCA activity assay, according to similar procedures described previously by Briggs et al. [31].

### PLN expression in mouse skeletal muscle

 To assess PLN levels in mouse skeletal muscle, soleus muscles were obtained from C57BL/6 mice and homogenized in buffer containing 250 mM glucose, 5 mM HEPES, 0.2 mM PMSF and 0.2% NaN_3_ (w/v). Proteins from human vastus lateralis and mouse soleus were electrophoretically separated on 13% gels using a Tricine-based system, transferred to either PVDF or nitrocellulose membranes and then probed for PLN following the same Western blotting procedures as described above. Soleus muscle from *Pln*-null mice [32] was used as a negative control. All mice were housed in an environmentally controlled room with a standard 12:12 light/dark cycle and allowed access to food and water *ad libitum*. All animal procedures were reviewed and approved by the Animal Care Committee of the University of Waterloo (AUPP 12-15) and are consistent with the guidelines established by the Canadian Council on Animal Care. 

### Statistical analysis

Data are presented as means ± SEM. Student’s *t*-test was used to compare the SLN, PLN, SERCA1a, and SERCA2a content in Type I fibers with that in Type IIA fibers, and to compare SERCA activity measurements made with and without the SLN antibody. *P* < 0.05 was considered significant.

## Results and Discussion

### SLN protein expression in human vastus lateralis

**Figure 1:**
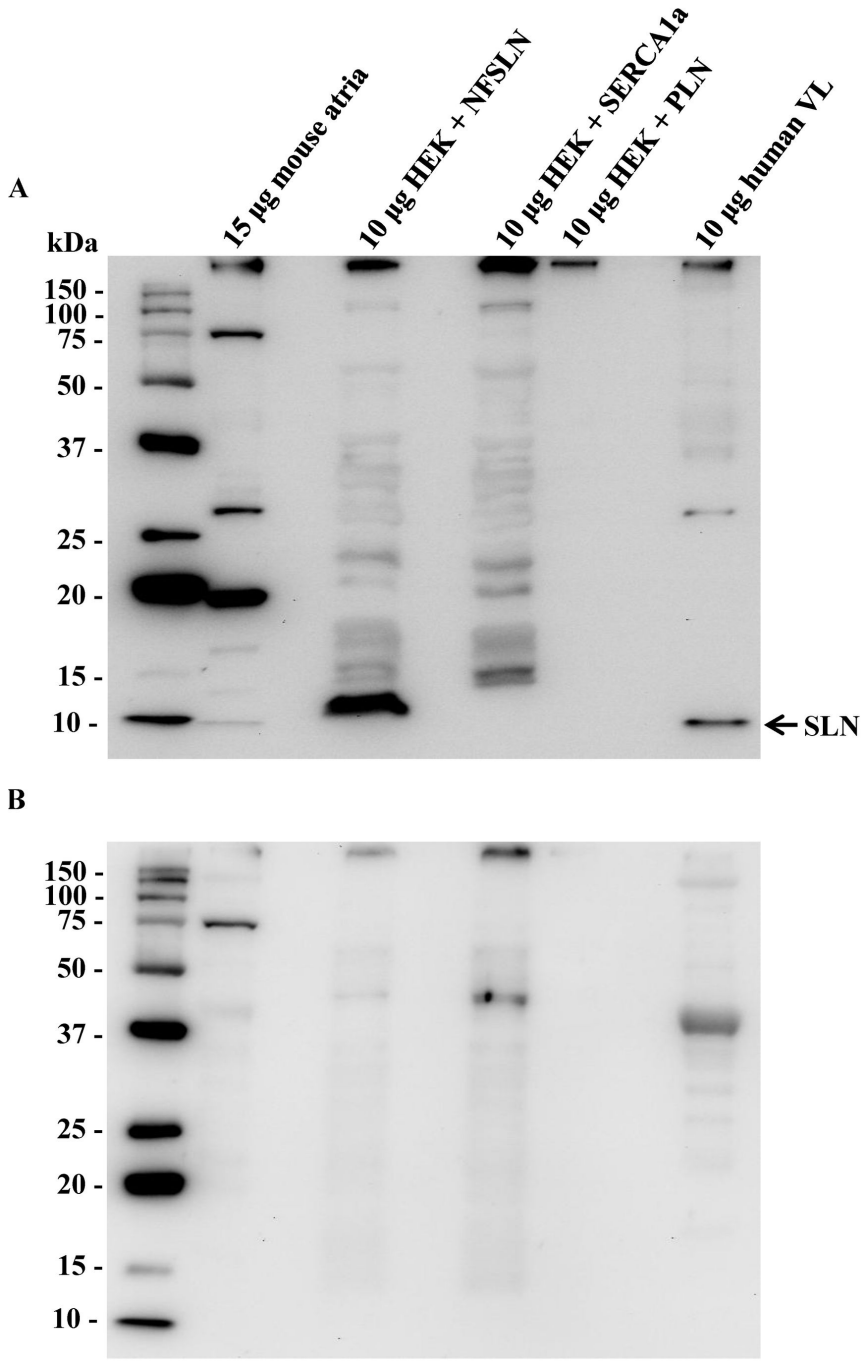
Sarcolipin expression in human vastus lateralis. A newly generated sarcolipin antibody was used to probe for sarcolipin protein expression in mouse atrial homogenate, HEK cells expressing flag-tagged SLN (HEK + NFSLN), HEK cells expressing SERCA 1a (HEK + SERCA1a), HEK cells expressing PLN (HEK + PLN), and human vastus lateralis (VL) without (**A**) and with (**B**) blocking peptide. *Left* *Lane*, 6 µL BioRad Precision Plus Western C^TM^ Molecular Weight Marker.

 Before assessing fiber-type co-expression of SERCA isoforms with SLN we first confirmed the presence of SLN protein in human skeletal muscle and determined the validity of a newly generated SLN antibody. An immunoreactive band for SLN was detected at approximately 10 kDa in the lanes containing mouse atrial homogenate, lysates from HEK-293 cells transfected with NF-SLN cDNA, and human vastus lateralis homogenate but not in lanes containing lysates from HEK-293 cells transfected with SERCA1a or PLN cDNAs (Figure 1A). The SLN antibody also labeled a prominent ~19 kDa protein band in mouse atria but not human vastus lateralis (Figure 1A). It is possible that this protein band could represent a SLN oligomer, which may exist in muscle membranes [33], or non-specific binding. In support of the latter, we have previously shown that this SLN antibody labels a ~19 kDa band in soleus homogenates from both WT and *Sln*-null mice [24]. The SLN signal for HEK-293 cells transfected with NF-SLN cDNA was detected at a slightly higher molecular weight due to the FLAG epitope attached to the N-terminus of SLN. Introduction of SLN blocking peptide to SLN antibody in a 5:1 ratio resulted in the elimination of the SLN immunoreactive band at 10 kDa (Figure 1B). Thus, we show for the first time that SLN protein is expressed in human skeletal muscle.

### Regulation of SERCA activity by SLN

**Figure 2:**
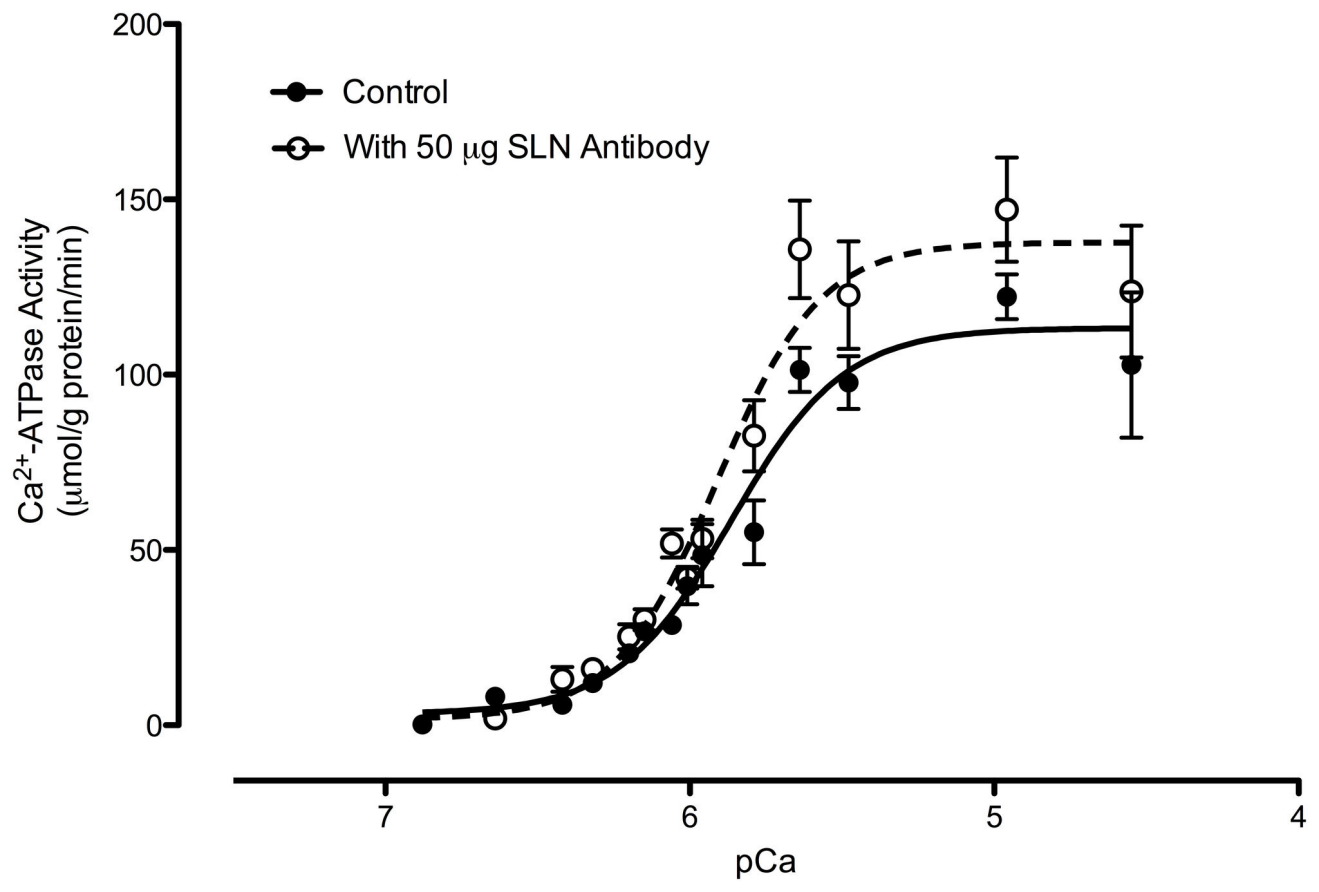
Ca^2+^ dependence of sarco(endo)plasmic reticulum Ca2+-ATPase (SERCA) activity. SERCA Ca^2+^-dependent ATPase activity was assessed in muscle homogenates from human vastus lateralis (n=4) with (open symbols) and without (Control, solid symbols) 50 μg of sarcolipin (SLN) antibody.

It is well established that the inhibitory function of PLN on SERCA activity in cardiac SR is abolished in the presence of a PLN monoclonal antibody [31,34,35]. Here, we assessed SLN inhibitory function in human vastus lateralis by measuring SERCA activity in the absence (control) and presence of the new SLN antibody. Consistent with the known effects of SLN on SERCA function [9,36], both maximal SERCA activity and the apparent SERCA affinity for Ca^2+^ were increased in the presence of the SLN antibody (Table 1 and Figure 2). Thus, regulation of SERCA pumps by SLN is consistent between cell, animal and human studies. 

**Table 1:** SERCA activity in human vastus lateralis.

	Vmax	*K*_Ca_, pCa	Δ*K*_Ca_
Control	122 ± 6.4	5.85 ± 0.02	--
With 50 μg SLN antibody	159 ± 11*	5.95 ± 0.02*	0.10

Values are means ± SEM. Homogenates from male human vastus lateralis (n=4) were analyzed for Ca^2+^-ATPase activity (μmol per g proteinmin**^*-*^** *^1^*) over Ca^2+^ concentrations ranging from *pCa* 6.9 to *pCa* 4.5. Vmax is the maximal SR Ca^2+^-ATPase activity; *K*_Ca_ is the negative logarithm of the Ca^2+^ concentration required to attain the half-maximal Ca^2+^-ATPase activity rate. *Significantly different (*P* < 0.05) from control.

### Fiber type-specific PLN and SLN co-localization with SERCA isoforms

**Figure 3:**
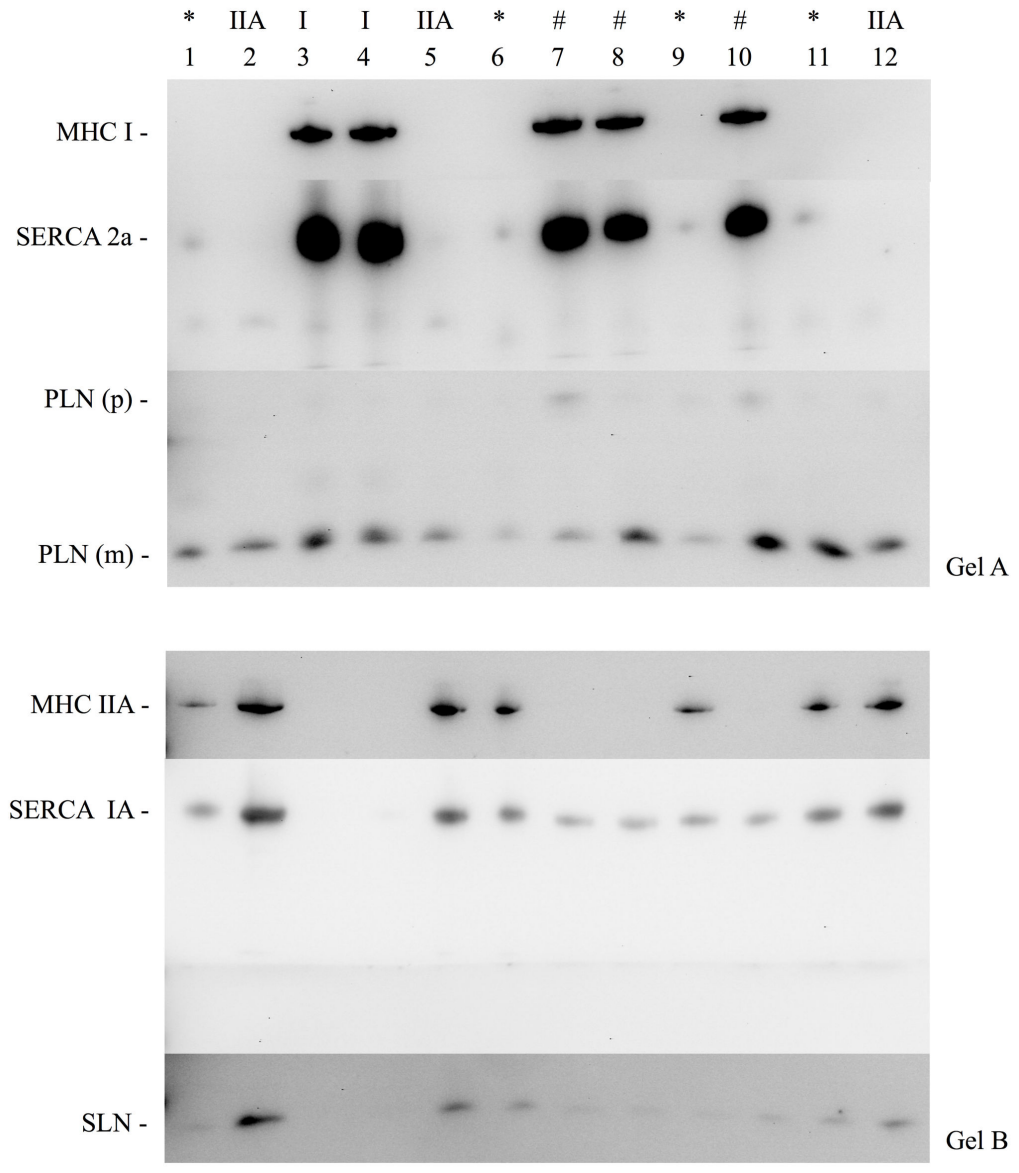
Representative Western blots on freeze-dried human single muscle fiber segments showing co-expression patterns of SERCA isoforms, MHC isoforms, sarcolipin and phospholamban. Solubilized single fibers were halved and loaded to either Gel A or Gel B. Typical co-expression of SERCA and MHC isoforms are seen in Type I (lane 3, 4), and Type IIA (lanes 2, 5, 12). #, Atypical co-expression of SERCA1a with MHCI isoform; *, Atypical co-expression of SERCA2a with MHCIIa isoforom; SLN, sarcolipin; PLN, phospholamban; p, pentamer; m, monomer.

**Figure 4:**
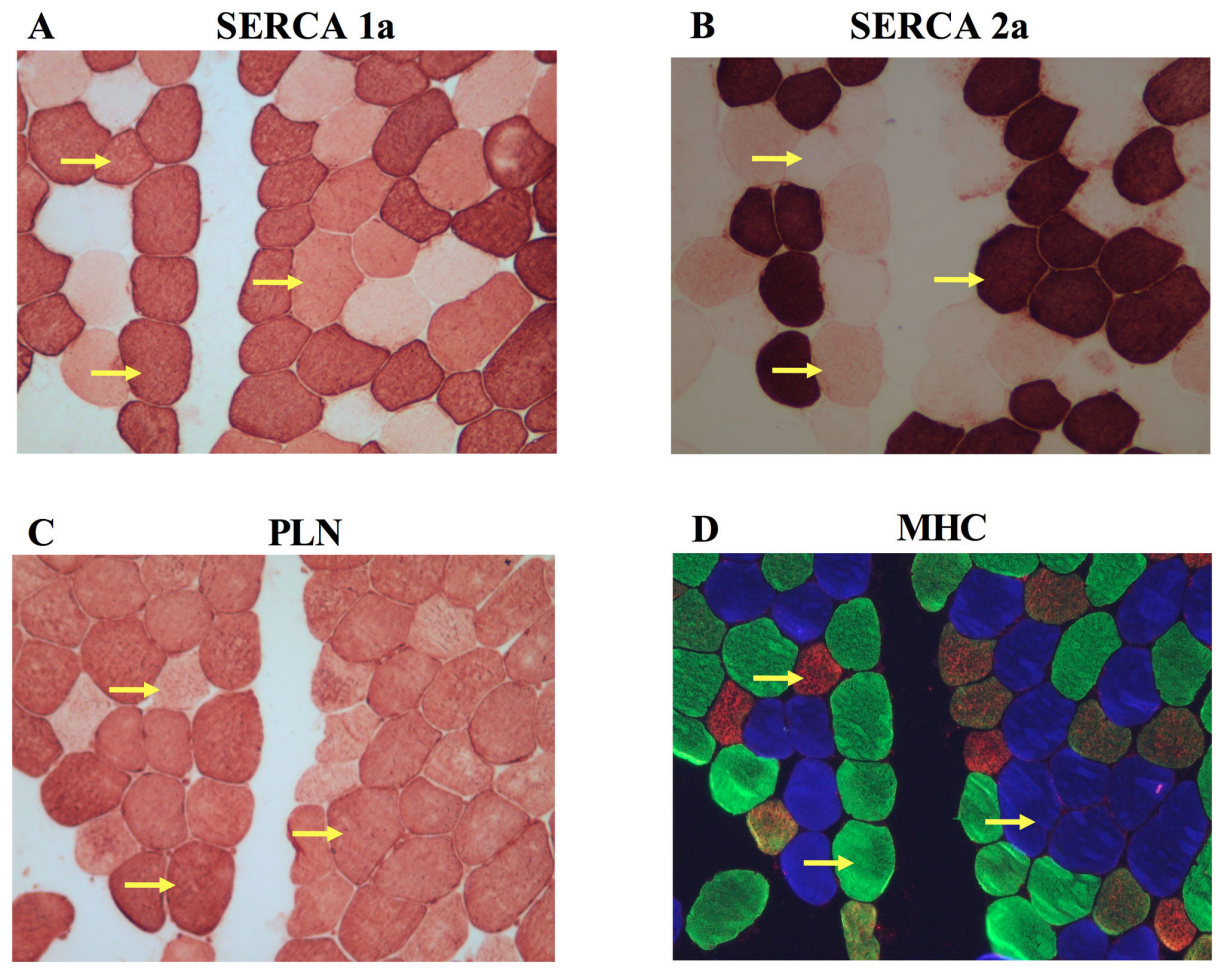
Representative immunohisto/fluorescent stains from human vastus lateralis muscle revealing co-expression patterns of SERCA1a, SERCA2a, and PLN with MHCI (blue), MHCIIa (green), and MHCIIx (red). Arrows highlight 3 different matched fibers showing typical co-expression of SERCA1a with MHCIIx, as well as atypical co-expression of SERCA1a with SERCA2a and MHCI and SERCA2a with SERCA1a and MHCIIa.

**Figure 5:**
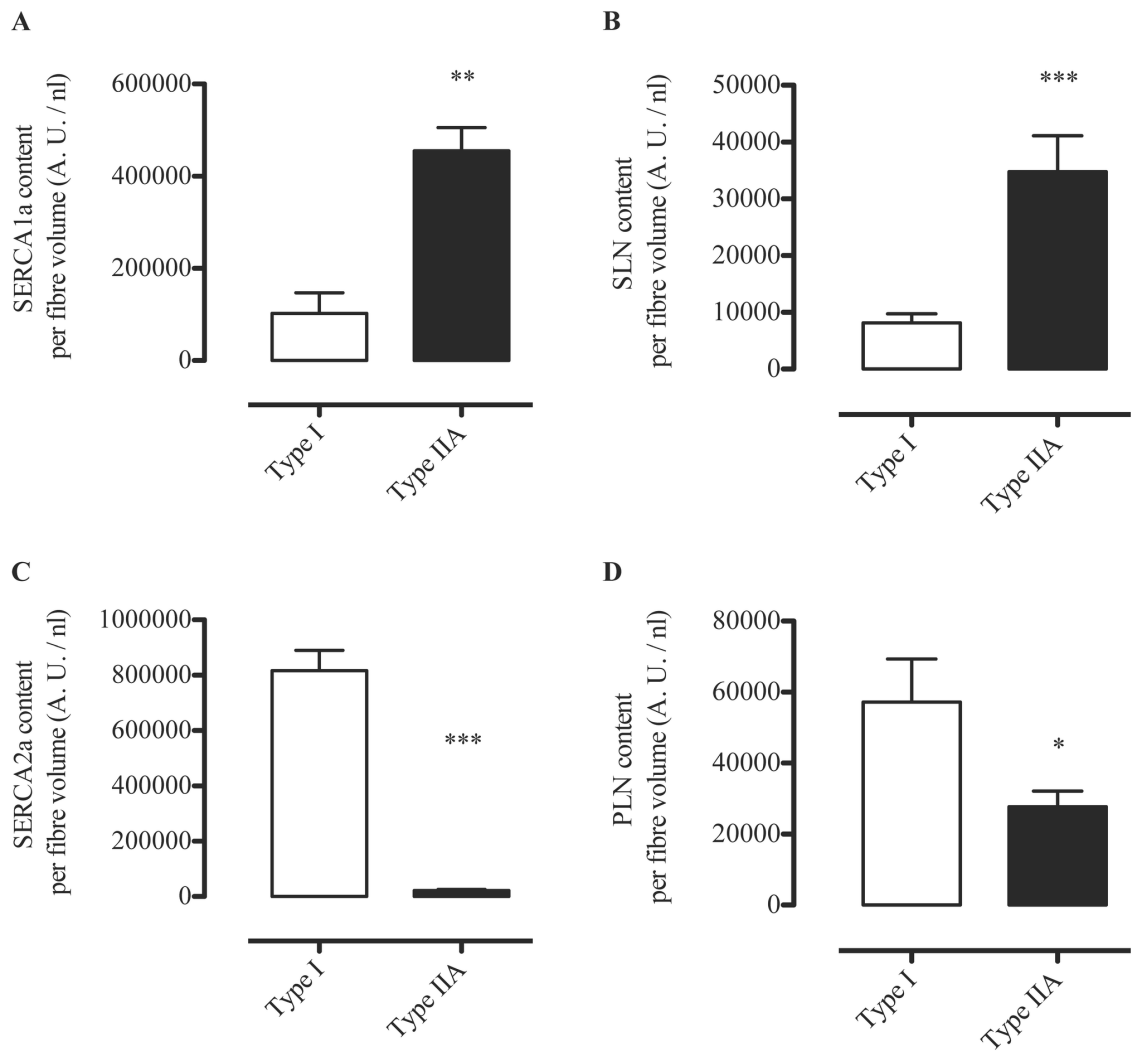
Results from semi-quantitative Western blot analyses showing average SERCA1a (A), SLN (B), SERCA2a (C) and PLN (D) optical density (arbitrary units /nl fiber volume) in Type I fibers vs Type IIA fibers. *, *p* < 0.05; **, *p* < 0.01; ***, *p* < 0.0001. For SLN and PLN, Satterthwaite *t*-value was used for analysis due to significantly different variances (SLN, *p* = 0.0007; PLN, *p* < 0.0001).

**Figure 6:**
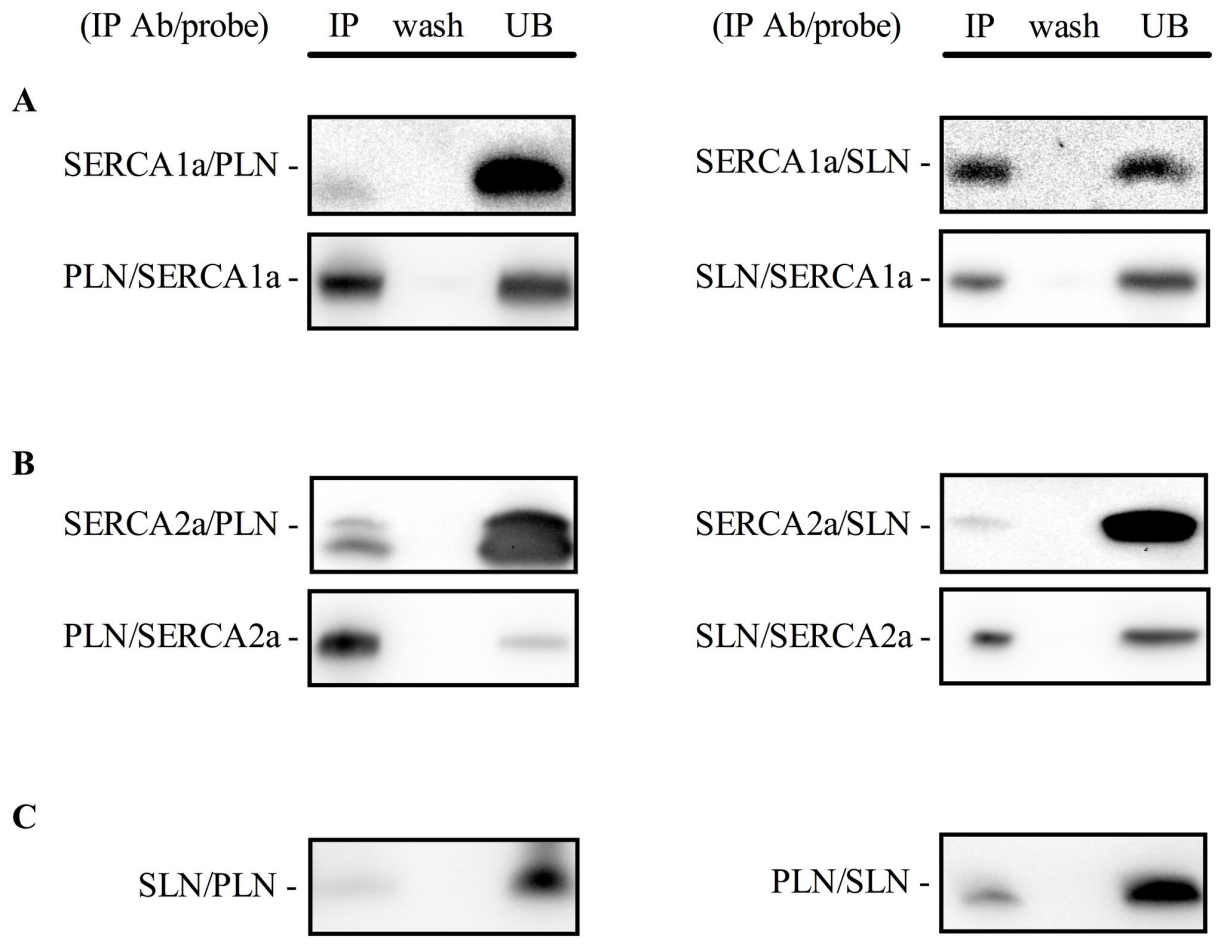
Co-immunoprecipitation of SERCA isoforms with sarcolipin and phospholamban. (A) SLN and PLN interactions with SERCA1a. (B) SLN and PLN interactions with SERCA2a. (C) SLN and PLN interactions with each other. IP Ab, immunoprecipitating antibody; IP, immunoprecipitate; wash, last wash before elution of immunoprecipitate; UB, unbound represents an aliquot (20 μl from 200 μl total volume) of the sample that did not immunoprecipitate using the IP Ab. Experiments were performed on human vastus lateralis muscle homogenates from 3 separate individuals.

It is unknown whether PLN and SLN regulate SERCA1a, SERCA2a or both SERCA isoforms in skeletal muscle. Evidence from animal and other human studies related to this issue is based on analyses on whole muscle homogenates and is, therefore, inconclusive, since SLN and PLN have been found to be expressed in muscles that express both SERCA isoforms [11,15,17]. To circumvent the limitation of using muscle homogenates, in this study we used single fiber Western blotting as previously described [5] with only a few modifications. By halving the solubilized protein obtained from the single fiber segments, performing electrophoresis on gradient gels, and strategically cutting PVDF membranes, we were able to probe for MHCI, SERCA2a, and PLN on gel/membrane A, as well as MHCIIa, SERCA1a, and SLN on gel/membrane B. SLN protein was detected only in fibers positive for SERCA1a, including those that had both SERCA1a and SERCA2a, irrespective of MHC isoform (Figure 3, Table 2). There were a small amount of fibers where SLN was not detected despite positive detection of SERCA1a (~3%). In contrast, PLN was detected in all single fibers analyzed with Western blotting regardless of SERCA isoform (co-expressed with SERCA1a and/or SERCA2a) or MHC isoform (co-expressed with MHCI and/or MHCIIa) (Figure 3, Table 2). These results were also confirmed by immunohisto/fluorescence staining demonstrating that PLN is localized in all human vastus lateralis fibers (Figure 4). Due to the non-specific binding of the SLN antibody (Figure 1A) we were unable to use the immunohisto/fluorescence technique to assess SLN co-localization. Our semi-quantitative densitometric analyses revealed that both SERCA1a (Figure 5A) and SLN (Figure 5 B) were expressed to a greater extent in MHCIIa compared with MHCI expressing fibers whereas SERCA2a (Figure 5C) and PLN (Figure 5D) expression were significantly higher in MHCI relative to MHCIIA expressing fibers. These results are, in part, consistent with our hypotheses since SLN and PLN expression seemingly follows that of SERCA1a and SERCA2a, respectively. Thus, these regulatory proteins may display preferential regulation of the SERCA isoforms physiologically; however, SLN and PLN each co-immunoprecipitated with both SERCA isoforms (Figure 6A and B), suggesting that both SLN and PLN can physically interact with and may regulate either SERCA isoform in human skeletal muscle. In fact, given that PLN was expressed in fibers that only expressed SERCA2a and also in fibers that only expressed SERCA1a, we conclude that PLN likely regulates both SERCA isoforms in human skeletal muscle. On the other hand, SLN was only detected in fibers if SERCA1a was also present so even though SLN co-immunoprecipitated with SERCA2a in muscle homogenates, we cannot say definitively that SLN regulates SERCA2a activity *in vivo*.

**Table 2:** Summary of sarcolipin and phospholamban co-localization with SERCA isoforms in single fibers obtained from human vastus lateralis.

	SERCA1a	SERCA2a	SERCA1a & SERCA2a
SLN	+	-	+
PLN	+	+	+

SLN, sarcolipin; PLN, phospholamban; SERCA, sarco(endo)plasmic reticulum Ca^2+^-ATPase.

### Co-expression of PLN and SLN

 A major finding in this study, which contradicted our hypothesis, was that SLN and PLN were co-expressed in fibers that either expressed SERCA1a alone or both SERCA1a + SERCA2a (Table 2). Nef et al. [37] reported that SERCA2a + PLN + SLN were co-expressed in human ventricular myocytes from patients with Tako-Tsubo cardiomyopathy and our study demonstrates for the first time that PLN and SLN are normally co-expressed with SERCA in healthy skeletal muscle. In fact, ~81% of fibers analyzed expressed both PLN and SLN which suggests that super-inhibition of SERCA1a or SERCA2a could be occurring physiologically, at least in human vastus lateralis. In support of this view, SLN was found to co-immunoprecipitate with PLN (Figure 6C) indicating that SLN and PLN may form a super-inhibitory ternary complex [38] with either SERCA isoform *in vivo*. Interestingly, the apparent affinity of SERCA for Ca^2+^ is much lower in human vastus lateralis (Table 1) compared with our previously reported values on mouse soleus [15]. PLN exists as a homopentamer and as a monomer [39,40], with scanning mutagenesis studies showing that the monomeric species is the active form capable of binding to and inhibiting SERCAs [40]. Superinhibition of SERCAs by SLN and PLN may be due to the ability of SLN to disrupt PLN pentamer structures thereby increasing the amount of PLN monomers capable of inhibiting SERCAs [41]. Mutations in either PLN or SLN that disrupt the PLN-SLN binary complex also reduce the extent of super-inhibition suggesting that tight binding between PLN and SLN is a prerequisite for ternary complex formation and super-inhibition [38]. It is possible that super-inhibition is a phenomenon that is only observed if non-physiological concentrations of PLN and SLN are examined *in vitro*. Nevertheless, our results point to the potential existence of super-inhibition of SERCAs in human skeletal muscle and future studies should examine this possibility. 

**Figure 7:**
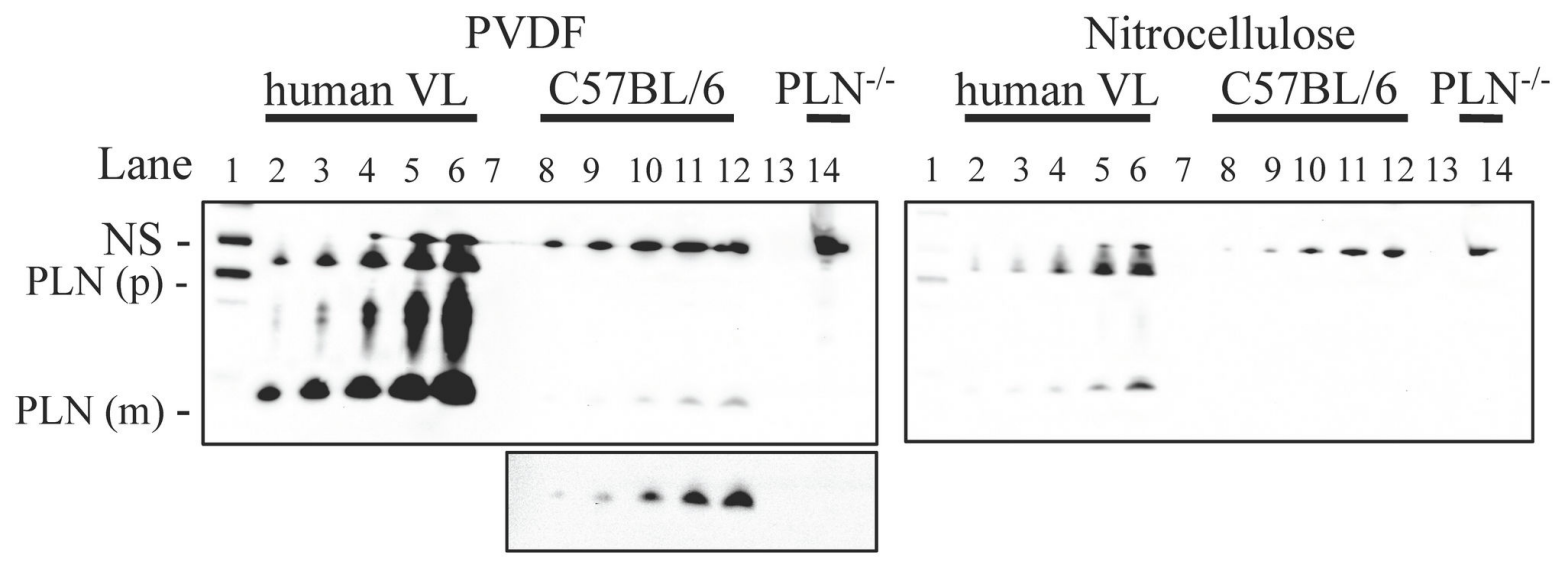
Phospholamban detection in human and mouse soleus muscle using PVDF or nitrocellulose membranes. Lane 1, PrecisionPlus^TM^ Western C Standards detected with StepTactin^TM^; Lane 2, 1 μg of human vastus lateralis (VL); Lane 3, 2.5 μg of human VL; Lane 4, 5 μg VL; Lane 5, 10 μg VL; Lane 6, 20 μg VL; Lane 7, empty; Lane 8, 2.5 μg of mouse (C57BL/6) soleus; Lane 9, 5 μg soleus; Lane 10; 12.5 μg soleus; Lane 11, 25 μg soleus; Lane 12, 50 μg soleus; Lane 13, empty; Lane 14, 50 μg PLN knock-out (*Pln^-/-^*) soleus. Luminata Forte^TM^ was used to detect both membranes with an exposure time of 4 min. PLN(m), monomer; PLN(p), pentamer; NS, non-specific. Inset below depicts PLN(m) for C57BL/6 and *Pln* ^*-/-*^ after adjusting brightness and contrast of image file.

In a recent report [15], we failed to detect PLN protein in mouse soleus, a skeletal muscle that contains relatively high levels of SLN protein [15,16,42], so we concluded that the potential for super-inhibition of SERCAs could be avoided in that tissue. Apparently, our inability to detect PLN in as much as 50 μg of mouse skeletal muscle total protein in our earlier study can be explained by the fact that we used nitrocellulose membranes for Western blotting because here, when we use PVDF membranes, we can detect PLN in as low as 2.5 - 5 μg of soleus total protein but could not detect PLN using nitrocellulose membranes (Figure 7). PLN signal detection in human vastus lateralis homogenate is also improved if proteins are transferred to PVDF compared with nitrocellulose membranes (Figure 7). Our current observations that PLN protein is expressed in mouse soleus are more in line with the reported changes in skeletal muscle phenotype caused by PLN ablation [43,44]. However, given the relatively low SLN and PLN protein contents found in mouse skeletal muscle, we have yet to be able to detect either SLN or PLN in single fibers obtained from mouse soleus despite the use of PVDF membranes. Therefore, we aim to further refine our Western blotting procedures to improve sensitivity of protein detection in order to determine the co-expression pattern of SLN and PLN with SERCA and MHC isoforms in single mouse skeletal muscle fibers.

### SERCA and MHC isoform co-expression

 Single fiber Western blotting has been used to show that SERCA1a is almost exclusively expressed in fast-twitch skeletal muscle fibers, whereas SERCA2a is expressed in slow-twitch fibers in both rats [45] and humans [5,45]. Using immunohistochemistry, we have also shown that SERCA1a is generally confined to fast-twitch fibers while SERCA2a is expressed in slow-twitch fibers in mouse skeletal muscle [15]. In that study, however, we detected SERCA2a in a small percentage of fast-twitch (Type IIA) fibers, but at much lower levels than in Type I fibers. Here, our human single fiber Western blot analyses also showed that SERCA2a is expressed in some fast-twitch fibers (Figure 3, lanes 1,6,9,11) and, as already mentioned, SERCA1a was expressed in some slow-twitch fibers (Figure 3, lanes 7,8,10). Immunohisto/fluorescence analyses confirmed the results found in single fiber Western blots (Figure 4). As summarized in Table 3, SERCA2a and SERCA1a were co-expressed in ~58% of the fibers that stained positive for MHCI and ~23% of the MHCI fibers analyzed with Western blotting. In addition, both SERCA1a and SERCA2a were found in ~18% of the MHCIIa fibers analyzed with immunohisto/fluorescence staining, and ~14% of the MHCIIa fibers analyzed with Western blotting. To our knowledge, this level of mismatch between SERCA and MHC isoforms is the highest reported to date for normal adult human skeletal muscle. In another study by Talmadge and colleagues [46], it was reported, based on immunohistochemical analyses, that only 9% of MHCI fibers and 3% of MHCIIa fibers from vastus lateralis biopsies taken from healthy adult humans, expressed both SERCA isoforms. High levels of SERCA and MHC mismatch is generally thought of as atypical which can be seen in states of severe muscle disuse such as spinal cord injury [46], bed rest [47] and denervation [7]. Differences in SERCA and MHC isoform expression patterns between our study and previous studies [5,46] are most likely due to analytical differences and/or sensitivity of the specific antibodies that were used, particularly for the detection of SERCA1a. In our experience, the anti-SERCA1a A52 moncolonal antibody [23] is highly sensitive, capable of detecting pg levels of protein even with low antibody concentrations and short (1 hr) incubation times. Based on our current results, we propose that the regulation of SERCA and MHC isoforms in normal adult human skeletal muscle occurs, to some extent, independently. Perhaps the co-existence of both SERCA1a and SERCA2a in either fiber type may be advantageous in human skeletal muscle as it might offer more versatility in Ca^2+^ handling and it likely explains why deletion of the *ATP2a1* gene is lethal in mice [48] but not in humans [49]. 

**Table 3:** Percentage distributions of SERCA1a and SERCA2a in both Type I and Type II fibers obtained from human vastus lateralis.

	Type I		Type IIA		Type IIX	
	IHC stain	Western blot	IHC stain	Western blot	IHC stain	Western blot
SERCA1a	58.2 ± 7.5	22.6 ± 6.7	100	100	100	-
SERCA2a	100	97.7 ± 0.8	17.7 ± 9.5	14.3 ± 2.4	0	-

Values are means ± SEM. IHC, immunohisto/fluorescence stain

## Conclusions

 Using single fiber Western blotting and immunohisto/fluorescence staining techniques, we present several novel findings regarding the co-expression patterns of SLN and PLN with SERCA and MHC isoforms in human vastus lateralis fibers. Our results show that fast-twitch Type II fibers contain relatively high levels of SLN and SERCA1a proteins and relatively low levels of PLN protein. SERCA2a protein was also detected at relatively low levels in some (~14-18%) Type II fibers. Conversely, slow-twitch Type I fibers contain relatively high levels of PLN and SERCA2a proteins and some (at least 22%) Type I fibers also contain relatively low levels of SLN and SERCA1a proteins. Based on these results, we conclude that SLN and PLN likely preferentially regulate SERCA1a, and SERCA2a, respectively. However, given that PLN protein was detected in every fiber, including fibers that only contained SERCA1a, and that both SLN and PLN were observed to co-immunoprecipitate with both SERCA isoforms, we conclude that PLN likely also regulates SERCA1a and SLN may also regulate SERCA2a in human skeletal muscle. Another major finding of this study was that SLN and PLN are co-expressed in most fibers, which suggests that super-inhibition of SERCAs may be physiologically important in the regulation of intracellular Ca^2+^ in human skeletal muscle. Emerging evidence from mouse models suggests that the primary biological function of SLN is to regulate thermogenesis by SERCA pumps [24,42,50] but PLN may not serve the same function [51]. While the physiological role of PLN in skeletal muscle is still unknown, the relatively high level of expression of both PLN and SLN in human skeletal muscle would suggest that the physiological role of these proteins in skeletal muscle is even more important in humans. 
